# Insights Into Enhanced Complement Activation by Structures of Properdin and Its Complex With the C-Terminal Domain of C3b

**DOI:** 10.3389/fimmu.2019.02097

**Published:** 2019-09-04

**Authors:** Ramon M. van den Bos, Nicholas M. Pearce, Joke Granneman, T. Harma C. Brondijk, Piet Gros

**Affiliations:** Crystal and Structural Chemistry, Department of Chemistry, Faculty of Science, Bijvoet Center for Biomolecular Research, Utrecht University, Utrecht, Netherlands

**Keywords:** complement system, alternative pathway, properdin, convertase, C3b, factor B

## Abstract

Properdin enhances complement-mediated opsonization of targeted cells and particles for immune clearance. Properdin occurs as dimers, trimers and tetramers in human plasma, which recognize C3b-deposited surfaces, promote formation, and prolong the lifetime of C3bBb-enzyme complexes that convert C3 into C3b, thereby enhancing the complement-amplification loop. Here, we report crystal structures of monomerized properdin, which was produced by co-expression of separate N- and C-terminal constructs that yielded monomer-sized properdin complexes that stabilized C3bBb. Consistent with previous low-resolution X-ray and EM data, the crystal structures revealed ring-shaped arrangements that are formed by interactions between thrombospondin type-I repeat (TSR) domains 4 and 6 of one protomer interacting with the N-terminal domain (which adopts a short transforming-growth factor B binding protein-like fold) and domain TSR1 of a second protomer, respectively. Next, a structure of monomerized properdin in complex with the C-terminal domain of C3b showed that properdin-domain TSR5 binds along the C-terminal α-helix of C3b, while two loops, one from domain TSR5 and one from TSR6, extend and fold around the C3b C-terminus like stirrups. This suggests a mechanistic model in which these TSR5 and TSR6 “stirrups” bridge interactions between C3b and factor B or its fragment Bb, and thereby enhance formation of C3bB pro-convertases and stabilize C3bBb convertases. In addition, properdin TSR6 would sterically block binding of the protease factor I to C3b, thus limiting C3b proteolytic degradation. The presence of a valine instead of a third tryptophan in the canonical Trp-ladder of TSR domains in TSR4 allows a remarkable ca. 60°-domain bending motion of TSR4. Together with variable positioning of TSR2 and, putatively, TSR3, this explains the conformational flexibility required for properdin to form dimers, trimers, and tetramers. In conclusion, the results indicate that binding avidity of oligomeric properdin is needed to distinguish surface-deposited C3b molecules from soluble C3b or C3 and suggest that properdin-mediated interactions bridging C3b-B and C3b-Bb enhance affinity, thus promoting convertase formation and stabilization. These mechanisms explain the enhancement of complement-mediated opsonization of targeted cells and particle for immune clearance.

## Introduction

Complement plays an important role in humoral immune responses against invading microbes, clearance of apoptotic cells and debris, and modulation of adaptive immune responses (1, 2). Initiation of the complement cascades through either the classical, lectin or alternative pathway converges in the formation of C3 convertase complexes, consisting of C3b and protease fragment Bb forming C3bBb, which generates a positive-feedback loop that amplifies the complement cascade yielding massive deposition of C3b onto the targeted surface. At this critical step, the complement system is heavily regulated. Intrinsically, the non-covalent C3bBb enzyme dissociates irreversibly into its components C3b and Bb with a half-life time of 1–2 min (3, 4). Host regulators, such as factor H (FH), decay-accelerating factor (DAF), and membrane-cofactor protein (MCP), provide protection of host cells against complement attack (5). FH and DAF inactivate the C3 convertase by promoting dissociation of C3bBb into C3b and Bb (5). FH and MCP have cofactor activity that enables factor I (FI) to bind and cleave C3b into iC3b, rendering it inactive, and unable to form new convertases (5, 6).

Properdin is the only known intrinsic positive regulator of the complement system (7–9). Properdin stabilizes C3bBb, increasing the half-life of the enzyme complex 5- to 10-fold (10). In addition, it has been indicated that properdin accelerates formation of pro-convertases C3bB (11) and reduces C3b inactivation by FI (12, 13). Furthermore, it has been suggested that, for some bacterial surfaces, apoptotic/necrotic cells or renal epithelial cells, properdin can function as a pattern recognition molecule, forming an initiating platform for the alternative pathway (14–19), although others claim that properdin binding to surfaces depends on initial C3b deposition (20, 21). Properdin deficiency results in increased susceptibility to infection by *Neisseria meningitidis* (22), with high mortality rates compared to deficiency of protein components (C5–C9) of the terminal pathway (23). In addition, properdin deficiency has been associated with other diseases, such as otitis media and pneumonia, as reviewed in Chen et al. (23).

Human properdin is an oligomeric plasma protein that is present in serum at relatively low concentrations (4–25 μg/ml) (8), compared to other complement components [~1.2 mg/ml for C3 and ~0.6 mg/ml for factor B (FB)] (24). In contrast to most other complement proteins, properdin is not produced by the liver, but expressed locally by various immune cells including neutrophils, monocytes, and dendritic cells (23, 25, 26). Therefore, at sites of inflammation properdin concentrations might be considerably higher than serum concentration. In serum, properdin is predominantly found as dimers, trimers and tetramers in the percentage ratios of 26:54:20% (8), although a small amount of pentamers and hexamers are also found (13, 27). At physiological conditions, no exchange between the oligomeric states of properdin is observed (8), but higher order aggregates form upon freeze-thaw cycles (28). A properdin protomer consists of 442 amino-acid residues with a fully-glycosylated molecular weight of 53 kDa (29). Properdin forms seven domains, an N-terminal domain of unknown fold, followed by six thrombospondin type I repeats (TSR) domains (29). TSR domains consist of ~60 amino-acid residues and have a thin and elongated shape (30), formed by only three anti-parallel peptide chains. The TSR-fold is structurally stabilized by regions forming β-sheets, three conserved disulphide bonds and by a structural WRWRWR motif [also referred to as Trp-ladder (31)] that forms a stack of alternating tryptophans and arginines through π-cation interactions (30). The N-terminal domain has often been referred to as TSR0 (9, 13, 32, 33), despite missing the WRWRWR motif. Properdin is highly post-translationally modified, resulting in 14–17 C-mannosylated tryptophans, four O-linked glycans, and one N-linked glycan (34, 35). Negative-stain electron microscopy (EM) has shown that oligomeric properdin forms ring-shaped vertices connected by extended and flexible edges (13, 27). Based on EM images and TSR domain deletions, it has been proposed that the vertices consist of interlocking C- and N-terminal domains of properdin protomers and the edges consist of three bridging TSR domains from a single protomer (13, 27, 29). EM images indicate that each properdin vertex binds a single C3bBb complex (13). Higgens et al. (29) showed that domain deletions of properdin TSR domains 4 through 6 results in altered oligomerization and loss of function, whereas deletion of TSR3 has no significant effect on either oligomerization or properdin function. Pedersen et al. (32) introduced a proteolytic cleavage site between properdin-domains TSR3 and TSR4 and thereby generated single properdin vertices for crystallographic studies. A 6.0-Å resolution crystal structure of a single properdin vertex in complex with C3bBb (32) [that was stabilized by *S. aureus* inhibitor SCIN (36)] showed that properdin binds to the α-chain region of C3b, revealing density adjacent to the C-terminal C345c (CTC) domain of C3b. However, the resolution of the crystallographic data (PDB ID: 5M6W) did not allow atomic modeling of the cleaved properdin (Pc) fragment.

In this study, we present the production of monomerized properdin variants that stabilize C3bBb using co-expression of properdin N- and C-terminal fragments. We determined crystal structures of monomerized properdin and its complex with the CTC domain of C3/C3b with diffraction data up to 2.0- and 2.3-Å resolution, respectively. These structures reveal the fold of the properdin N-terminal domain, the properdin domain arrangement that yields the properdin ring-shaped vertex structure, stabilization of Trp-Arg interactions in the Trp-ladder provided by tryptophan C-mannosylation, structural flexibility of the TSR4 domain and functionally important extensions of the TSR5 and TSR6 domains. The structure of monomerized properdin in complex with the C3/C3b-CTC domain identifies the specific regions of properdin involved in binding FB and Bb that enhance pro-convertase formation and convertase stabilization, respectively. Finally, we propose a model for properdin oligomers stabilizing convertases on surfaces based on re-analysis of the 5M6W-diffraction data set.

## Materials and Methods

### Molecular Cloning and Construct Design

Human properdin (UniProtKB-P27918) cDNA was obtained from Open Biosystems (Dharmacon Inc.). Domain boundaries were chosen based on both UniProt assignment and crystal structures of thrombospondin I domains TSR2 and TSR3 (PDB ID: 1LSL) (30). In addition to full-length properdin (res. 28–469), four N-terminal constructs were created, P^N1^ (res. 28–132), P^N1^′ (res. 28–134), P^N12^ (res. 28–191), and P^N123^ (res. 28–255), comprising the first two, three, and four N-terminal domains of properdin; and P^456^ (res. 256–469) comprising the three C-terminal domains. The N-terminal domain boundary of the C3/C3b-CTC domain (res. 1517–1663) was chosen based on the structure of C3b [PDB ID: 5FO7 (37)]. All inserts were generated by PCR using clone specific primers that include a 5′ BamHI restriction site that results in an N-terminal Gly-Ser cloning scar in all constructs and a NotI restriction site at the 3′ end of the insert. The NotI site results in a C-terminal extension of three alanine's in all constructs, except for P^N12^ and C3/C3b-CTC, where a stop codon was introduced prior to the NotI site. All inserts were cloned into pUPE expression vectors (U-Protein Express BV, Utrecht, the Netherlands). For small-scale (4 ml) expression tests, one of the constructs (either the N- or C-terminal fragment) included a 6x-His purification tag. In large-scale co-expressions P^456^ included a C-terminal 6xHis-tag, with no tag on the N-terminal constructs. Similarly, constructs for full-length properdin included a C-terminal 6xHis-tag and the C3/C3b-CTC construct contained an N-terminal 6xHis-tag.

Recombinant proteins were transiently expressed in Epstein-Barr virus nuclear antigen I (EBNA1)- expressing, HEK293 cells (HEK293-EBNA) (U-Protein Express BV, Utrecht, the Netherlands). For crystallization purposes, proteins were expressed in GnTI^−^ HEK293-EBNA cells. N-terminal and C-terminal properdin fragments were co-expressed using a 1:1 DNA ratio. Cells were grown in suspension culture at 37°C for 6 days post-transfection. For each culture, supernatant was collected by a low-speed spin (1,000 × g for 10 min), followed by a high-speed spin (4,000 × g for 10 min) to remove any remaining cell debris. Subsequently, 3 ml/L Ni-Sepharose Excel beads (GE Healthcare) was added to the supernatant and the mixture was incubated for 2 to 16 h with constant agitation at 4°C. The beads were washed with 10-column volumes of buffer A (20 mM HEPES pH 7.8, 500 mM NaCl) and 10 column volumes of buffer A supplemented with 10 mM imidazole. Bound protein was subsequently eluted with buffer A supplemented with 250 mM imidazole. For small-scale (4 ml) expression tests of properdin fragments no further purification steps were performed, whilst for large-scale (1 L) expressions, pooled fractions were concentrated, and further purified by size-exclusion chromatography (SEC). P^N12/456^ for SPR was purified with a Superdex 200 16/600 (GE Healthcare) using 25 mM HEPES pH 7.8, 150 mM NaCl as the running buffer. All other properdin complexes were purified on a Superdex 200 10/300 Increase (GE Healthcare) column using either 20 mM HEPES pH 7.4, 150 mM NaCl (properdin, P^N1/456^, P^N1^′/456) or 25 mM HEPES pH 7.8 with 100 mM NaCl (P^N12/456^ for crystallizations) as the running buffer. The C3/C3b-CTC domain was purified on a Superdex 200 16/600 (GE Healthcare) in 20 mM HEPES pH 7.4, 150 mM NaCl. Human wild type FB, catalytically inactive (S699A) double-gain-of-function (D279G, N285D) FB mutant (FB^dgf‡^) (38), factor D (FD), DAF1-4 and Salp20 were purified as described previously (39–41). C3 and C3b were purified from human plasma as described in Wu et al. (40). Full-length properdin was stored at 4°C and all other proteins were flash frozen by plunging into liquid N_2_ and stored at −80°C.

### C3 Convertase Stability Assay

To generate C3 convertase, purified C3b (obtained after cleavage of human serum-derived C3) was mixed with catalytically inactive FB^dgf‡^ at a ratio of 1:1.1 in the presence of 5 mM MgCl_2_. After incubation for 5 min at 37°C, FD was added to a ratio of C3bB:FD of 1:0.1 and the mixture was incubated for another 5 min at 37°C, after which the C3 convertase was stored on ice till further use. C3 convertase was diluted to 1.5 μM with ice-cold buffer (20 mM HEPES pH 7.4, 150 mM NaCl and 5 mM MgCl_2_). Either 6 μM P^N1/456^ or P^N12/456^ or an equal volume of buffer (control) was added to the C3 convertase in a ratio of 1:2 resulting in a final concentration of 2 μM P^N1/456^ or P^N12/456^ and 1 μM C3 convertase. The mixture was incubated at 37°C for 1 h and subsequently put on ice. The amount of C3 convertase was analyzed by analytical SEC using a Superdex 200 10/300 Increase pre-equilibrated with 20 mM HEPES pH 7.4, 150 mM NaCl, and 5 mM MgCl_2_ at 18°C on a Shimadzu FPLC.

### Surface-Plasmon Resonance

C3b was generated from C3 through the addition of FB and FD to a C3:FB:FD molar ratio of 1:0.5:0.03 in the presence of 5 mM MgCl_2_ and incubation at 37°C. At 10 min intervals fresh FB was added to ensure complete conversion of C3 to C3b. Subsequently, C3b was biotinylated on the free cysteine that is generated after hydrolysis of the reactive thioester (42); EZ-Link Maleimide-PEG2-Biotin (Thermofisher) was added to a final concentration of 1 mM to the freshly produced C3b (13 μM) and the mixture was incubated for 3 h at room temperature. C3b was separated from unreacted Biotin-Peg2-Maleimide by SEC using a S200 10/300 increase column pre-equilibrated in 20 mM HEPES pH 7.4, 150 mM NaCl. Purity and conversion of C3 to C3b were analyzed by SDS-PAGE. To analyze equilibrium binding to monomerized properdin, we used P^N1^′/456 that includes an additional Cys-Pro (res. 133–134) at the C-terminus of TSR1. P^N1^′/456 (45 μM) was biotinylated as described for C3b and separated from excess biotin with a 5 mL HiTrap Desalting column (GE Healthcare) pre-equilibrated in 20 mM HEPES pH 7.4, 150 mM NaCl. Biotinylated proteins were spotted on a SensEye P-Strep chip (SensEye) at 50 nM for 60 min with a continuous flow microspotter (CFM, Wasatch). Equilibrium binding kinetics were analyzed using an IBIS-MX96 (IBIS Technologies). All experiments were performed in 20 mM HEPES pH 7.4, 0.005% (v/v) Tween-20, 150 mM NaCl at 4 μl/s. For experiments involving C3bB and C3bBb the buffer was supplemented with 5 mM MgCl_2_. Analyses at low ionic strength were performed at a NaCl concentration of 50 mM. Analytes were injected from low to high concentration in 14 2-fold incremental steps. In equilibrium binding analyses involving C3bB or C3bBb, C3bB was generated on a C3b coated SPR surface by injecting 100 nM FB^dgf‡^ for 5 min prior to each analyte injection. C3bBb was generated from C3bB by injections of 100 nM FD for 5 min after each FB injection. Where indicated, DAF1-4 (1 μM), FD (100 nM), and/or Salp20 [1 μM for experiments with C3b alone and 5 μM in experiments with C3 (pro) convertase] were injected, to regenerate the C3b surface. Salp20, a properdin inhibitor from deer tick (43), was required to dissociate full length properdin from C3b. In all experiments, the SPR surface was washed with buffer supplemented with 1 M NaCl at 8 μl/s for 30 s at the end of each cycle. Temperature was kept constant at 25°C. Prism (GraphPad) was used for data analysis. K_D_′s were determined by fitting the end point data to $Y=Bmax*\frac{X}{KD+X}+Background$.

### Crystallization, Data Collection, and Structure Determination

P^N1/456^ and the C3/C3b-CTC domain were dialyzed overnight at 4°C using a 3.5 kDa cutoff Slide-A-Lyzer Mini Dialysis Unit (Thermo Scientific) against 10 mM HEPES, 50 mM NaCl, pH 7.4. The N-linked glycan on Asn428 of P^N1/456^ was removed by including 1% v/v EndoHF (New England BioLabs) during the dialysis. P^N1/456^, P^N12/456^, and the C3/C3b-CTC domain were concentrated to 8.7 mg/ml, 10 mg/ml, and 10.3 mg/ml, respectively.

Crystals were obtained using the sitting drop vapor diffusion method at 18°C. Crystals of P^N12/456^ were grown in 100 mM sodium citrate pH 5.5 and 20% (w/v) PEG 3,000 and cryoprotected by soaking in mother liquor supplemented with 25% (v/v) glycerol. Crystals of P^N1/456^ were grown in 0.2 M potassium sulfate and 20% (w/v) PEG 3350, and cryoprotected by soaking in mother liquor supplemented with 25% (v/v) ethylene glycol. P^N1/456^ and C3/C3b-CTC were mixed in a 1:1 molar ratio at 8 mg/ml and crystals were grown in 8% v/v Tacsimate pH 5.0 and 20% (w/v) PEG 3350, and cryoprotected by soaking in mother liquor supplemented with 25% glycerol. After harvesting, crystals were cryo-cooled by plunge freezing in liquid N_2_. All diffraction data were collected at the European Synchrotron Radiation Facility (ESRF) on beamlines ID29 (P^N12/456^) and ID23-1 (P^N1/456^-CTC, P^N1/456^). The diffraction images were processed with DIALS (44) and the integrated reflection data were then anisotropically truncated with the STARANISO web server (45).

Structures were solved by molecular replacement using Phaser (46). Atomic models were optimized by alternating between refinement using REFMAC (47), and manual building in Coot (48). C- and O-linked glycosylation restraints were generated within Coot, using ACEDRG (49). The structure of P^N12/456^ was refined with restraints generated from P^N1/456^ using ProSMART (50). Data was deposited at the RSCB Protein Data Bank (51) with PDB IDs 6S08, 6S0A, and 6S0B. We also re-analyzed data deposited for Pc in complex with C3bBb-SCIN (32) (PDB ID: 5M6W). An initial position for the properdin molecule was obtained by superposing our P^N1/456^-CTC model onto the CTC domain of the two C3b molecules in the model deposited by Pedersen et al. (32). Subsequently, TSR2 from P^N12/456^ was added and manually adjusted to fit the density. In addition, for one of the two copies in the asymmetric unit, density corresponding to TSR3 was apparent. A TSR model derived from TSR2 from thrombospondin-1 [PDB ID: 1LSL (30)], containing the canonical cysteines and Trp-ladder residues, but otherwise consisting of poly-alanines, was placed into this density. The resulting model was further refined using the LORESTR refinement pipeline (52). Coordinates of the re-refined properdin-C3bBb-SCIN complex (PDB ID: 5M6W) are available from the authors upon request.

## Results

### Production of Monomerized Properdin by Co-expression of N- and C-Terminal Fragments

**Figure 1:**
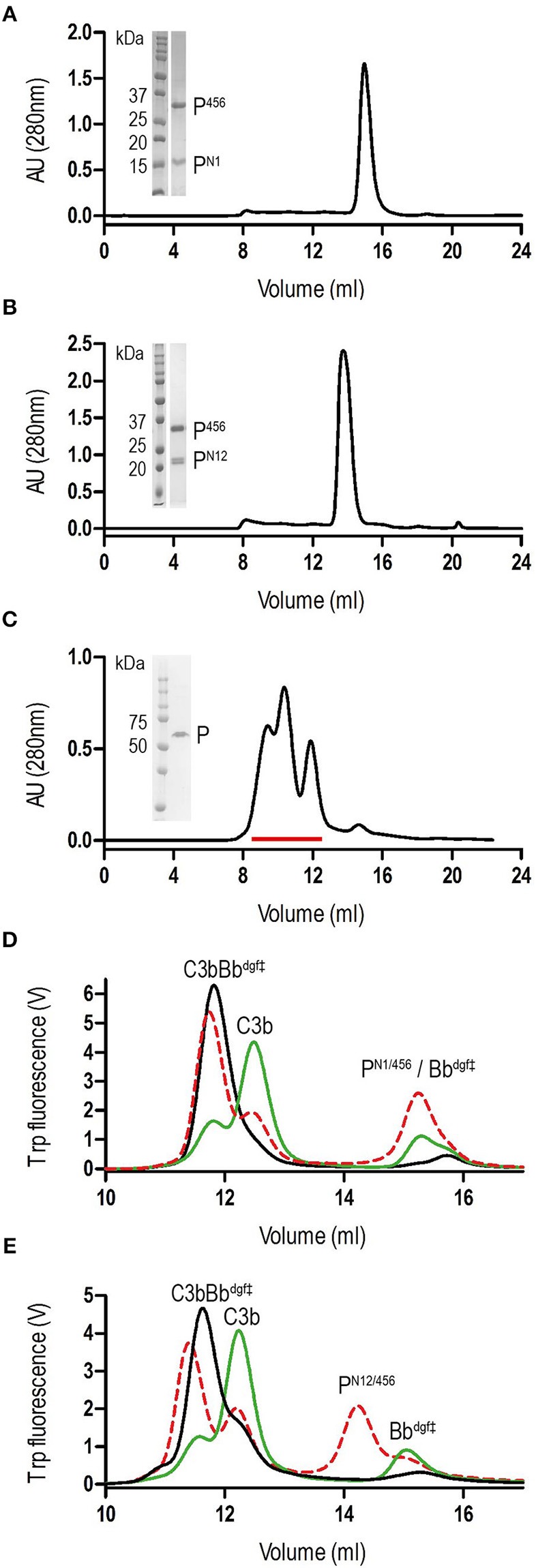
Purification of properdin and stabilization of C3 convertase. **(A)** SEC elution profile and SDS-page of P^N1/456^. **(B)** SEC elution profile and SDS-page of P^N12/456^. **(C)** SEC elution profile and SDS-page of properdin, pooled fractions are indicated by the red line. **(D)** SEC elution profiles of C3 convertase (C3bBb^dgf‡^) incubated in the presence (red dashed line) or absence (green line) of P^N1/456^ for 1 h at 37°C compared to the sample at *t* = 0 (black line). **(E)** As panel **(D)** but with P^N12/456^ instead of P^N1/456^.

We generated N-terminal constructs of properdin, comprising the N-terminal domain of unknown fold and TSR1, TSR2, and TSR3, denoted P^N1^ (res. 28–132), P^N12^ (res. 28–191), and P^N123^ (res. 28–255), and a C-terminal construct comprising TSR4, TSR5, and TSR6, P^456^ (res. 256–469). Small scale expression of isolated His_6_-tagged terminal fragments followed by IMAC-affinity purification resulted in no significant expression of P^456^, whereas co-expression of N- and C-terminal fragments yielded both fragments in ~1:1 ratio in all cases. We therefore decided to continue with large-scale co-expression of the two shorter N-terminal fragments, P^N1^ and P^N12^, with P^456^ with the latter carrying a C-terminal His_6_-tag (see section Materials and Methods). IMAC-affinity purification yielded stable protein complexes consistent with one-to-one non-covalent complexes of P^N1^ with P^456^ and P^N12^ with P^456^, denoted P^N1/456^ and P^N12/456^, respectively. Both P^N1/456^ and P^N12/456^ yielded monodisperse peaks during size-exclusion chromatography (SEC) consistent with a single monomerized species (Figures 1A,B), whereas recombinant full-length properdin produced a SEC spectrum with multiple peaks consistent with a mixture of dimeric, trimeric, and tetrameric properdin (Figure 1C). Large-scale expression and purification of P^N1/456^ and P^N12/456^ yielded ca. 5-8 mg per liter culture.

### Monomerized Properdin Binds and Stabilizes C3 Convertases

Stabilization of C3 convertases was analyzed by monitoring the decay of pre-formed C3bBb in the presence and absence of properdin (Figures 1D,E). In the absence of properdin, ~75% of the C3bBb^dgf‡^ was dissociated into C3b and Bb^dgf‡^ after 1 h at 37°C, whereas in the presence of P^N1/456^ or P^N12/456^ dissociation of C3bBb^dgf‡^ was reduced to ~20–25%, indicating that P^N1/456^ and P^N12/456^ stabilized C3 convertase to a similar extent.

**Figure 2:**
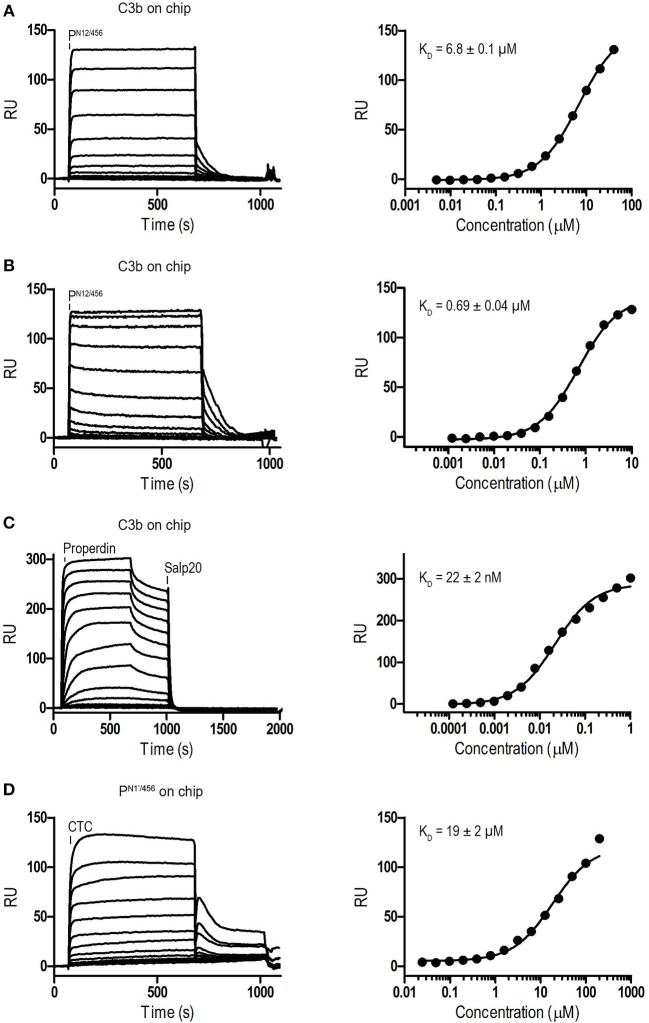
Surface Plasmon Resonance (SPR) analysis showing interaction of properdin with C3b and the C3b/C3 CTC domain. SPR sensorgrams (left) and equilibrium binding plots (right). **(A)** Interaction of P^N12/456^ (concentration range: 4.9 × 10^−3^ μM to 40 μM) with a C3b coated chip at physiological ionic strength (150 mM NaCl). **(B)** interaction of P^N12/456^ (concentration range: 1.2 × 10^−3^ μM to 10 μM) with a C3b coated chip at low ionic strength (50 mM NaCl). **(C)** Interaction of properdin (concentration range: 1.2 × 10^−4^ μM to 1 μM) with a C3b coated chip. **(D)** Binding of the C3/C3b CTC domain (concentration range: 2.4 × 10^−2^ μM to 200 μM) to a P^N1^′/456 coated chip. The data point at 200 μM C3/C3b CTC was considered as an outlier and was not used to determine the K_D_. Where indicated Salp20 was used to regenerate the surface.

**Figure 3:**
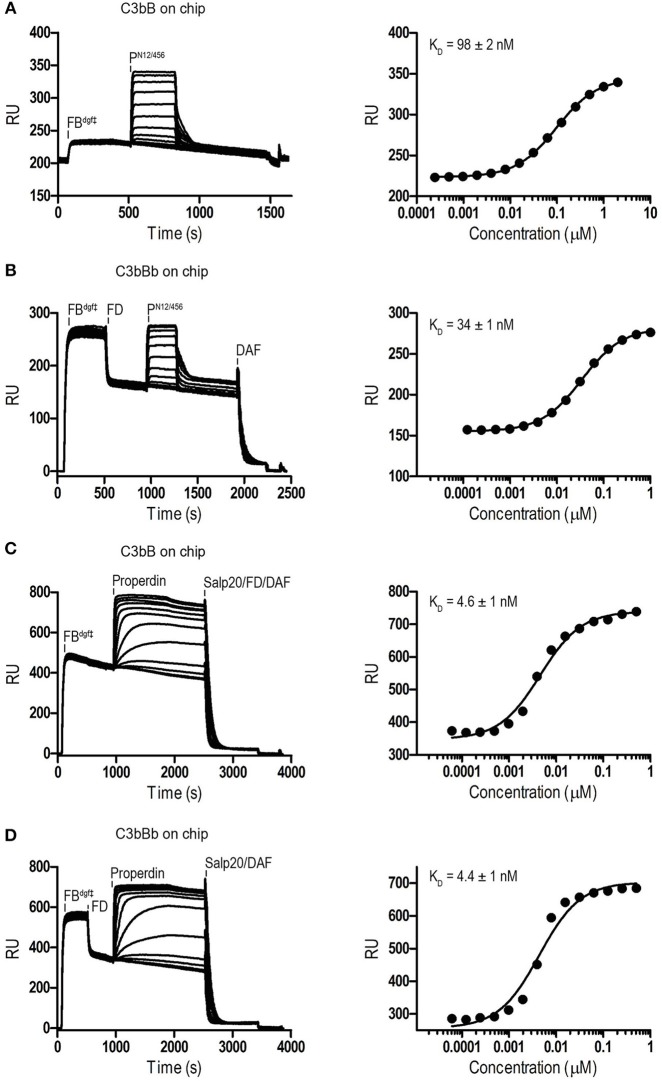
SPR analysis showing interaction of properdin with C3 pro-convertase. SPR sensorgrams (left) and equilibrium binding plots (right). C3bB^dgf‡^ and C3bBb^dgf‡^ were generated on the chip by injecting FB^dgf‡^ or FB^dgf‡^ and subsequently FD on a C3b coated chip. **(A)** Interaction of P^N12/456^ (concentration range: 2.4 × 10^−4^ μM to 2 μM) with C3bB^dgf‡^. **(B)** Interaction of P^N12/456^ (concentration range: 1.2 × 10^−4^ μM to 1 μM) with C3bBb^dgf‡^. **(C)** Interaction of properdin (concentration range: 6.1 × 10^−5^ μM to 0.5 μM) with C3bB^dgf‡^. **(D)** Interaction of properdin (concentration range: 6.1 × 10^−5^ μM to 0.5 μM) with C3bBb^dgf‡^. Where indicated Salp20, FD, and DAF were used to regenerate the surface.

Binding affinities of P^N12/456^ for C3b, pro-convertase C3bB and convertase C3bBb were determined using surface plasmon resonance (SPR) equilibrium binding experiments. C3b was biotinylated at its reactive thioester, which allows coupling to streptavidin-coated SPR sensor chips in an orientation reflecting that of surface bound C3b. Under physiological salt conditions, P^N12/456^ bound C3b with a K_D_ of 6.8 ± 0.2 μM, which is similar to the K_D_ of 7.8 μM reported by Pedersen et al. for single properdin vertices generated by proteolytic cleavage (32), but much lower than the apparent K_D_ of 22 ± 2 nM for oligomeric properdin (Figure 2). At low ionic strength (50 mM NaCl), interaction between P^N12/456^ and C3b appeared much stronger with a K_D_ of 0.69 ± 0.04 μM. Next, we generated pro-convertases C3bB and convertases C3bBb on the chip (see section Materials and Methods). P^N12/456^ bound C3bB and C3bBb with a K_D_ of 98 ± 2 nM and 34 ± 1 nM, respectively, (Figure 3), whereas properdin oligomers bound with an apparent K_D_ of 4.6 ± 1 nM and 4.4 ± 1 nM, respectively. Thus, P^N12/456^ binds to C3b, C3bB and C3bBb (in order of increasing affinity).

Previous data (13, 32) suggested that the main interaction site of properdin with C3b is localized on the C3b-CTC domain. Therefore, we analyzed binding of the isolated C3/C3b-CTC domain to a P^N1^′/456 coated SPR chip. The C3/C3b-CTC domain binds P^N1^′/456 with a K_D_ of 18.6 ± 1.6 μM, which is comparable to the K_D_ of 6.8 ± 0.2 μM we observed for C3b and P^N12/456^, suggesting that the primary binding interface of C3b is indeed provided by the CTC domain (Figure 2). Overall, these data indicated that the non-covalent complexes P^N1/456^ and P^N12/456^ bound C3b and stabilized C3bBb similar to an excised monomeric version of full-length oligomeric properdin.

### Structure Determination of Monomerized Properdin and Its Complex With C3/C3b-CTC

P^N1/456^ and P^N12/456^ crystallized as thin plates, and resulted in highly anisotropic data, with anisotropic resolution limits of 2.0–2.9 Å and 2.5–3.9 Å, respectively. P^N1/456^ in complex with C3/C3b-CTC crystallized as long rods and pyramids. While the pyramid-shaped crystals showed poor diffraction, P^N1/456^-CTC rod-shaped crystals diffracted anisotropically with resolution limits of 2.3–2.7 Å. Data collection statistics are shown in Table 1.

**Table 1:** Diffraction-data collection and refinement statistics.

	**P^**N1/456**^-CTC**	**P^**N1/456**^**	**P^**N12/456**^**
Wavelength (Å)	0.9789	0.9789	0.9763
Resolution range	63.08–2.31 (2.60–2.31)	79.61–2.03 (2.31–2.03)	102.88–2.52 (2.71–2.52)
Space group	P 21 21 21	C 1 2 1	I 4 2 2
**Cell dimensions**			
a, b, c (Å)	71.47, 71.50, 134.18	112.00, 114.86, 39.82	114.75, 114.75, 232.26
α, β, γ (°)	90, 90, 90	90, 99.56, 90	90, 90, 90
Total reflections	113,523 (7,011)	60,691 (2,967)	142,792 (8,720)
Unique reflections	21,145 (1,538)	17,424 (871)	16,212 (810)
Multiplicity	5.4 (4.6)	3.5 (3.4)	8.8 (10.8)
Completeness (Spherical)	68.6 (17.1)	54.5 (8.7)	60.5 (15.5)
Completeness (ellipsoidal)	90.3 (71.1)	89.5 (61.1)	93.6 (71.3)
Diffraction limits and eigenvectors of ellipsoid fitted to diffraction cut-off surface: (Å)	a^*^: 2.705 b^*^: 2.647 c^*^: 2.287	0.952 a^*^-0.307 c^*^: 2.027 b^*^: 2.289 0.926 a^*^+ 0.377 c^*^: 2.979	a^*^: 2.515 b^*^: 2.515 c^*^: 3.914
Mean I/sigma(I)	11.1 (1.5)	3.4 (1.5)	7.5 (1.5)
Wilson B-factor (Å^2^)	47.02	23.66	53.29
R-merge	0.100 (1.267)	0.161 (1.249)	0.185 (1.639)
R-pim	0.047 (0.637)	0.100 (0.784)	0.089 (0.726)
CC1/2	0.996 (0.560)	0.982 (0.469)	0.996 (0.629)
Reflections used in refinement	21,137	17,421	16,209
R-work/R-free	0.230/0.277	0.212/0.248	0.248/0.267
Number of non-hydrogen atoms	3,645	2,589	2,906
Macromolecules	3,452	2,343	2,705
Ligands	123	130	190
Solvent	70	116	11
RMS (bonds) (Å)/(angles) (°)	0.014/1.82	0.014/1.87	0.014/1.97
Ramachandran outliers (%)	0.67	0	0.55
Rotamer outliers (%)	3.65	2.38	7.42
Clashscore	2.80	5.92	8.24
Average ADP (Å^2^)	52.83	29.76	64.02
Macromolecules	52.88	29.62	63.25
Ligands	58.33	34.92	76.76
Solvent	40.23	26.87	32.57

Values in parentheses are for reflections in the highest resolution shell. The

* *denotes reciprocal space*.

**Figure 4:**
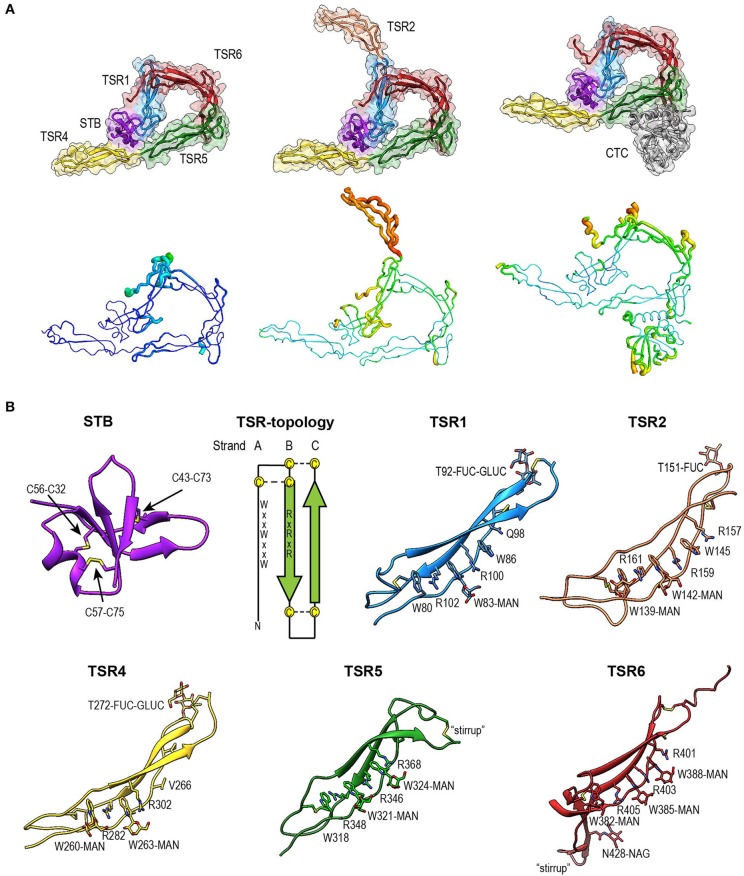
Overview of properdin structures. **(A)** From left to right: P^N1/456^, P^N12/456^, and P^N1/456^-CTC. Structures are depicted in cartoon representation with a semi-transparent molecular surface (top row) and as ADP cartoon putty (bottom row). ADP colors for all three structures are on the same scale of 10–140 Å^2^. **(B)** Cartoon representation of individual properdin domains; the TSR Trp-ladder residues, disulphides, and glycans are depicted as sticks. A schematic representation of the general TSR domain topology is included, showing the three strands and the position of the WxxWxxW and RxRxR motifs; the three disulphides are represented by dashed lines. TSR domains are shown with the Trp-ladder in approximately the same orientation. TSR1 and TSR2 were taken from FP^N12/456^, TSR6 from FP^N1/456^-CTC and STB, TSR4 & TSR5 from FP^N1/456^. Unless stated otherwise, domains are colored as follows: STB (purple), TSR1 (blue), TSR2 (coral), TSR4 (yellow), TSR5 (green), TSR6 (red) from properdin, and the C3/C3b CTC domain (gray).

We first determined the crystal structure of P^N1/456^ in complex with C3/C3b-CTC using the C3b-CTC domain [PDB ID: 5FO7 (37)] as a search model for molecular replacement with Phaser (46). A minimal TSR model was generated with Sculptor (53) using a sequence alignment (54) of TSR1, TSR4, TSR5, and TSR6 in combination with TSR2 from thrombospondin-1 [PDB ID: 1LSL (30)]. This model was then used in subsequent rounds of molecular replacement, which resulted in the positioning of TSR1, 4, 5, and half of TSR6 accounting for ~80% of the total structure. The N-terminal domain and the remaining part of TSR6 were built using Coot (48). Structure determination continued with further rounds of model building (48) and structure refinement (47), until convergence. The refined model of P^N1/456^ taken from P^N1/456^-CTC was used in molecular replacement to solve the structures of P^N1/456^ and P^N12/456^. After initial placement, P^N12/456^ was completed by molecular replacement using the TSR model. Model refinement statistics for all structures are listed in Table 1 final models are shown in Figure 4.

### Fold of the Properdin N-Terminal Domain

The crystal structure of properdin revealed that the N-terminal domain (res. 28–76) adopts a compact globular fold, containing two β-sheets and a single α-helix stabilized by three disulphide bonds (Figure 4B). A homology search using the Dali server (55) indicated that the properdin N-terminal domain is most closely related to transforming growth factor β binding protein-like (TB) domains; the closest structural homologs for the properdin N-terminal domain are the TB domains of human follistatin (PDB ID 5JHW, chain C/D, Dali z-score 5.9), and follistatin-like 3 (PDB ID 3B4V chain H, Dali z-score 5.6) and the hyb2 and TB4 domains of human Fibrillin-1 (respectively: PDB ID 2W86, Dali z-score 5.4; PDB ID: 1UZQ, Dali z-score 5.2) (56). TB domains are characterized by 8 cysteines resulting in a 1–3, 2–6, 4–7 and, though not always present, 5–8 disulphide pattern, where the 5–8 disulphide links the domain core to the C-terminal tail and Cys3, 4, and 5 form a characteristic triple cysteine motif (57, 58). The properdin N-terminal domain contains three disulphides that match the 1–3, 2–6, 4–7 disulphides of the TB core and misses the 5–8 disulphide and connecting C-terminal tail. We refer to this as the short TB (STB) fold.

### Properdin-TSR Domains

Five of the six TSR domains of properdin are present in the structures of P^N1/456^, P^N12/456^, and P^N1/456^-CTC (Figure 4). The TSR domains of properdin display minor to major variations from the TSR domain fold as described for the structures of TSR2 and 3 from thrombospondin-1 (30); these are shown schematically in Figure 4B.

**Figure 5:**
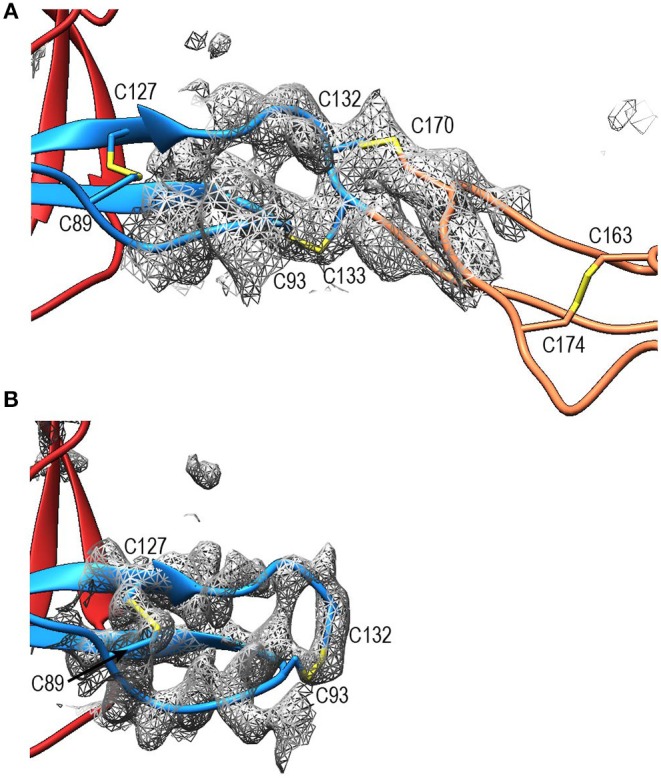
Disulphides at the properdin TSR1-TSR2 interface. **(A)** Cartoon representation of the TSR1-TSR2 interface in P^N12/456^ with disulphides represented as sticks. The terminal Cys133 of TSR1 forms the canonical disulphide with C93 in theTSR1 A-B loop, whereas Cys132 forms a disulphide with Cys170 in the B-C loop of TSR2. **(B)** The TSR1 distal end in P^N1/456^ showing the “incorrect” disulphide between Cys132 and Cys93. Electron density is shown at 1-rmsd contour level. Colors are as follows: TSR1 (blue), TSR2 (coral), TSR6 (red), and disulphides are shown in yellow.

Compared to TSR2 and 3 from thrombospondin-1, properdin domain TSR1 (res. 77–133) lacks a five-residue β-bulge preceding β-strand C, referred to as “jar-handle” motif, that provides H-bonding interactions with the indole ring of the first tryptophan of the Trp-ladder. Instead of this β-bulge, the C-strand in TSR1 is extended by two residues and the typical H-bonding interactions of the β-bulge are substituted by Ser112 in the B-C loop, which is observed within H-bond distance of the Trp80 indole ring. In the TSR1-Trp ladder, a glutamine residue resides at the position of the third arginine, resulting in a lost π-cation interaction with the last Trp. Preceding the prototypical C-terminal Cys (Cys133), TSR1 contains an additional cysteine (Cys132) that connects to Cys170 of TSR2, as observed in the structure of P^N12/456^ (Figure 5A). However, our construct P^N1^ is terminated at Cys132. As a consequence, we observed a non-native disulphide bond between Cys93-Cys132 and increased disorder at the C-terminal end of TSR1 in the structure of P^N1/456^ and P^N1/456^-CTC (Figure 5B); however, the overall fold of TSR1 was not affected.

TSR2 (res. 134–191) displayed the consensus TSR fold, with only minor deviations besides the additional cysteine (Cys170).However, this domain was not well-defined by the density as reflected by its high atomic displacement parameters (ADP) (Figure 4A).

**Figure 6:**
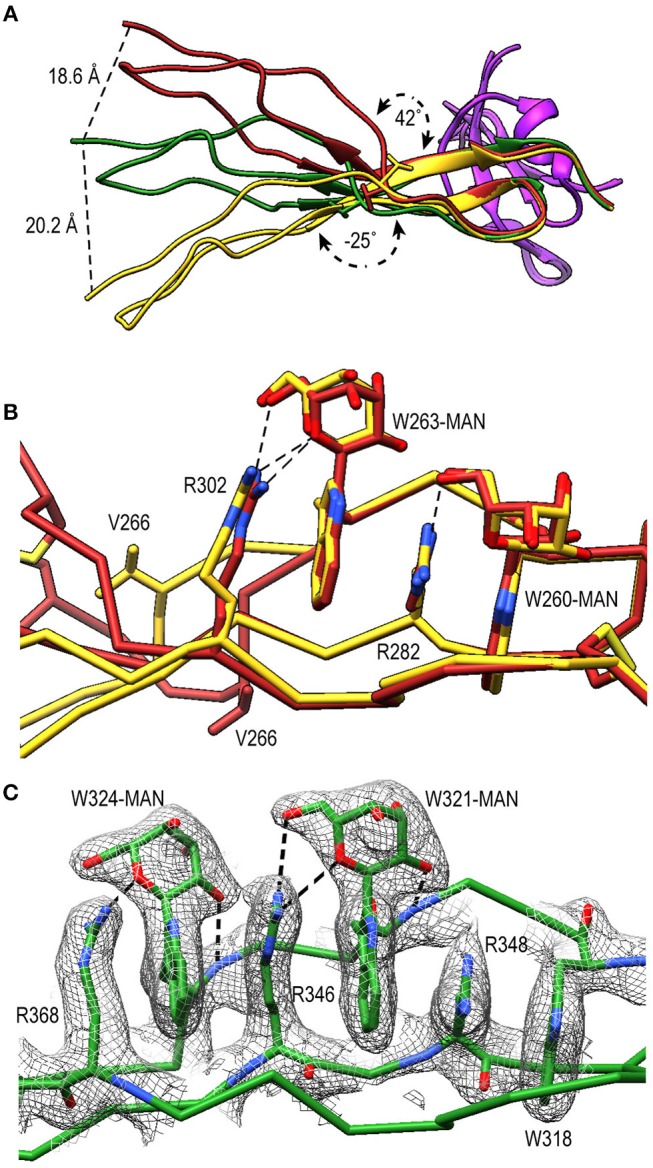
A shorter Trp-ladder allows structural flexibility of TSR4. **(A)** Cartoon representation of TSR4 from P^N1/456^ (yellow), P^N12/456^ (red), and P^N1/456^-CTC (green). Structures were superposed using the STB domain, shown in purple, as reference. The TSR4 domain is bent at the position of V266 (shown in sticks) resulting in distances of 18.6 Å, 20.2 Å, and 28.3 Å between the Cα-atoms of the N-terminal residues (S255) in each of the three models. The angle between the proximal (residues 256–266 and 279–303) and distal (residues 267–278 and 304–312) parts of TSR4 are indicated. **(B)** Trp-ladder residues in TSR4 from P^N1/456^ (yellow) and P^N12/456^ (red) showing the distortion of the TSR domain at the position of the missing third tryptophan, which is replaced by Val266. **(C)** Trp-ladder residues in TSR5 (green) shown as representative for a prototypical TSR Trp-ladder, The TSR-fold is stabilized by mannosyl-Trp/Arg H-bonds. Trp-ladder residues are shown in sticks and electron density is shown at 1-rmsd contour level. H-bonds are indicated as dashed lines.

TSR4 (res. 256–312) showed striking variations in the structures of P^N1/456^, P^N12/456^, and P^N1/456^-CTC (Figures 6A,B). In the Trp-ladder of TSR4 the canonical third tryptophan is replaced by a valine (Val266). A comparison of TSR4 from all three structures shows that TSR4 displays a bending-like motion at this position (Figures 6A,B). The distal part of TSR4 is held in place by interaction with the STB domain, but the proximal part, where the short Trp-ladder, comprising Trp260 and Trp263 in strand A, Arg282 in strand B and Arg302 in strand C, is located is at a different position in each of the three structures resulting in a distance of 28.3 Å between the Cα atoms of TSR4 Ser255 in P^N1/456^ and P^N12/456^.

TSR5 (res. 313–376) displayed well-defined electron density in all three structures and closely resembled the TSR-consensus fold. However, the canonical third arginine in strand B of TSR5 is replaced by Gln344. Gln344 forms a H-bond with Arg368 from strand C and Arg364 is in π-cation stacking conformation with Trp324. Thus, the stacking of Trp-ladder residues is effectively conserved. The most striking feature of TSR5 is a six-residue insertion (res. 328–333) (29), in the A-B loop between Cys327 and Cys337 that forms a loop that protrudes from the TSR domain.

**Figure 7:**
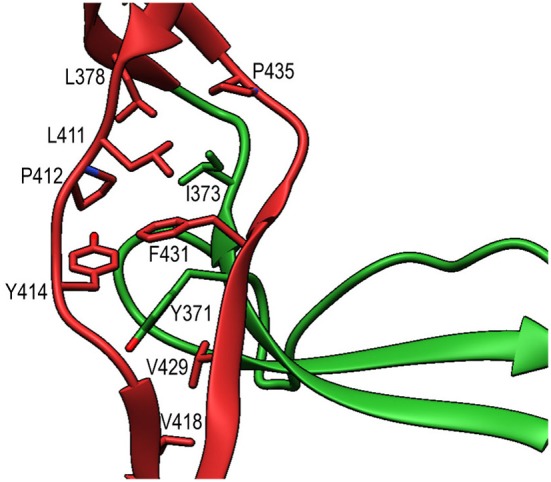
Interactions between TSR5 and TSR6 stabilize the TSR6 β-hairpin. Cartoon representation of the TSR5/TSR6 (green/red) interface with residues that form the hydrophobic core that stabilizes the TSR6 β-hairpin shown in sticks.

TSR6 (res. 377–469) showed a larger deviation from the typical TSR-fold and has a boomerang-like appearance, due to a 22 residue-long insertion (res. 412–434) (29) in the B-C loop. This insertion forms a β-hairpin loop that protrudes from the TSR6-core (Figure 4B). The core part of TSR6 makes an angle of 147° with TSR5, pointing toward TSR1, and the TSR6 β-hairpin protrudes at a 70° angle from the domain core toward and beyond TSR5. Residues 430–438 from the TSR6 β-hairpin are part of a β-sheet with the end of strand C from TSR5 (Figure 7). A hydrophobic core consisting of Pro435, Tyr371, and Ile373 from TSR5 and Leu378, Leu411, Pro412, Tyr414, Val418, Val429, and Phe431 from TSR6 stabilizes the base of the β-hairpin. Similar to TSR1, TSR6 lacks a “jar-handle” motif. In this case, the jar-handle H-bonding interactions are substituted by the backbone carbonyl from Glu440 in the B-C loop, which forms a H-bond with NH1 of the first Trp, Trp382, of the Trp-ladder. In TSR6 Arg405 is not stabilized by a residue from strand C and both Arg405 and Trp382 are not in a π-cation stacking conformation and thus do not contribute to the stability of the Trp-ladder.

### Properdin Glycosylation

The tryptophans of TSR Trp-ladders are typically C-type mannosylated, where the C1 of an α-mannose is attached to the C2 in the indole ring of the Trp (34, 35, 59). We could clearly identify C-mannosylation for 11 out of 14 Trp-ladder tryptophans (Figure 4B). For the majority of these, we observe that the O2 oxygen of the mannosyl-Trp moiety interacts with its backbone nitrogen, whereas the O5 and O6 oxygens form H-bonds with the side chain of the adjacent Arg, which further stabilizes the TSR domain fold (Figures 6B,C). In addition to C-mannosylation, TSR domains usually display O-linked glycosylation of a Thr or Ser residue that precedes the cysteine in loop A-B (35, 60, 61). This glycosylation constitutes the attachment of a β-glucose-1,3-α-fucose glycan through a linkage between the C1 atom of the fucose and the Thr or Ser side chain oxygen (61). In P^N12/456^, we observe O-fucosylation of TSR1 (Thr92), TSR2 (Thr151), and TSR4 (Thr272) (Figure 4B), although the TSR2 glycan is poorly defined. In all structures, the O-fucosylation of TSR4 is especially well-defined and is involved in properdin oligomerization, as described below. Finally, we observe N-glycosylation of Asn428, which is located in the B-C loop insertion in TSR6 and has been shown not to be important in properdin function (29).

### Properdin Oligomerization

A previously reported model for properdin oligomerization described the properdin vertex as a ring formed by four TSR domains each comprising a quarter of the ring (13) and formed by two inter protomer contacts (13, 27). The structures of P^N1/456^ and P^N12/456^ showed that the properdin vertex consists of the STB domain, TSR1, part of TSR4, TSR5, and TSR6 domains. These domains form a ring-like structure through interfaces formed by the STB and TSR1 domains with TSR4 and TSR6, respectively. TSR2 and ~66% of TSR4 are protruding from the vertex and form the properdin edges along with TSR3, which is absent in P^N1/456^ and P^N12/456^. The boomerang-shaped TSR6 forms approximately half of the ring, with an extensive interface between the distal end of TSR6 and TSR1, and the long insertion in the B-C loop of TSR6 locked firmly in place by interactions with TSR5 (Figure 7).

**Figure 8:**
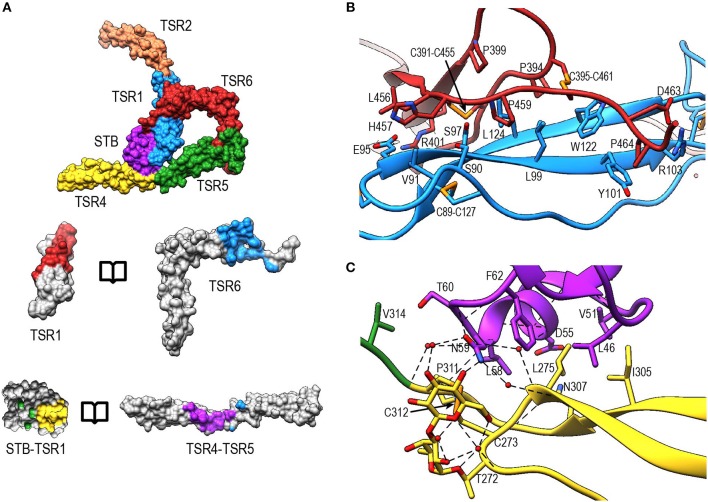
Properdin inter-protomer interfaces. **(A)** (top) Surface representation of P^N12/456^ colored by individual domains: STB (purple), TSR1 (blue), TSR2 (coral), TSR4 (yellow), TSR5 (green), and TSR6 (red). (bottom) Individual TSR domains with atoms colored by interaction with the opposing protomer (contacts defined as atoms within 5 Å). **(B)** Interactions between TSR1 and TSR6 with key residues involved in the interaction shown in stick representations. **(C)** As panel **(B)** for interactions between STB and TSR4/5. For clarity disulphides are colored orange.

The interface between TSR6 and TSR1 is formed by the distal end of TSR6, which includes the A-B loop and the C-terminal region of strand C, and the β-sheet of TSR1 (Figures 8A,B). This interface is predominantly mediated by hydrophobic interactions, involving residues Leu99, Tyr101, Trp122, and Leu124 from TSR1 and Pro399, Pro459, Pro464, Cys391-Cys455, and Cys395-Cys461 from TSR6. In addition, hydrogen bonds are formed between the backbone atoms of Leu124 from TSR1 and Cys391 in TSR6, respectively, and between sidechains of Ser90 and Ser97 and the backbone carbonyl of His457 and Leu456, respectively. Additionally, salt bridges are formed between Glu95 and Arg103 in TSR1 and Arg401 and Asp463 in TSR6. The interaction between Glu95 and Arg401 is not visible in P^N1/456^-CTC since the region containing Glu95 is not well-defined in this structure.

The second interface between properdin protomers is formed by the STB domain and TSR4 (Figures 8A,B). This interface is characterized by a hydrophobic core involving Leu47, Val51, Leu58, Phe62 from the STB domain and Leu275, Ile305, and Pro311 in TSR4. In addition, there are hydrophilic interactions between Asp55 and the backbone carbonyl moiety of Leu58 from the STB domain and Asn307 and the backbone nitrogen of Cys312 of TSR4, respectively. The O-linked glycan on Thr272 from TSR4 contributes directly to the interaction via a hydrogen bond with Asn59 on the STB domain as well as multiple water-mediated interactions.

### Interaction of Properdin With C3b

**Figure 9:**
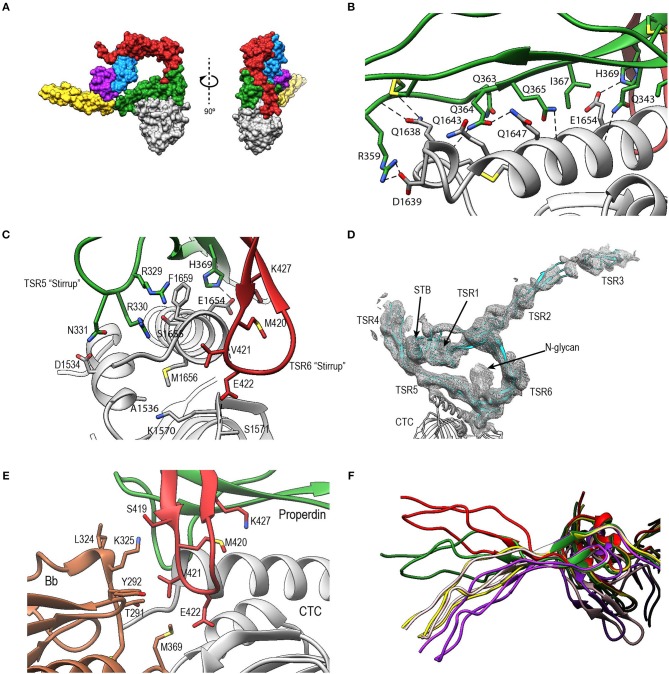
Properdin-convertase interactions. **(A)** Surface representation of P^N1/456^-CTC. Domains colored as follows: STB (purple), TSR1 (blue), TSR4 (yellow), TSR5 (green), TSR6 (red) from properdin, the C3/C3b CTC domain (gray), and Bb (brown). **(B)** Detailed view of the interaction between TSR5 and the C3/C3b CTC C-terminal α-helix. **(C)** Side view of P^N1/456^-CTC, 90° rotated compared to **(B)** showing details of the interaction between the TSR5 and TSR6 stirrup loops and C3/C3b-CTC. In **(B,C)** proteins are shown in cartoon representation with side chains of key residues that are involved in the interaction shown in sticks. H-bonds are indicated as dashed lines. **(D)** Detail of the properdin-C3bBb-SCIN complex showing electron density at 1-rmsd contour level. **(E)** Close-up of the properdin-C3b-Bb interface showing the two properdin stirrup-loops that are sandwiched between C3b and Bb. Putative interaction in the properdin-C3bBb interface are shown as sticks. **(F)** TSR4 from all five properdin structures that are described in this paper: P^N1/456^ (yellow), P^N12/456^ (red), P^N1/456^-CTC (green), and two copies of properdin in the properdin-C3bBb-SCIN complex (purple and pink) with models superposed using the distal part of TSR4 (residues 267–278 and 304–312).

In P^N1/456^-CTC, properdin-TSR5 sits on top of the C3/C3b-CTC domain with an ~angle of 20° between the main body of TSR5 and the C3/C3b-CTC C-terminal α-helix (Figure 9A). This interface is characterized by mainly hydrophilic interactions, involving TSR5 residues Gln343, Gln363, Gln364, His369, and C3/C3b-CTC residues Gln1638, Gln1643, and Glu1654, and a salt bridge between TSR5 Arg359 and C3/C3b-CTC Asp1639 (Figure 9B). The C-terminal end of the C3/C3b-CTC α-helix is embraced by two loops, which resemble stirrups, formed by the insertions in the core structure of properdin TSR5 and TSR6, respectively (TSR5 res. 328–333 and TSR 6 res. 419–426) (Figure 9C). The TSR6 stirrup is partially disordered in the absence of C3/C3b-CTC, but well-defined in the P^N1/456^-CTC complex. The two “stirrups” provide additional properdin-C3b interactions; the TSR5 stirrup interacts with C3/C3b-CTC through cation-π stacking of Arg329 with C3/C3b Phe1659 and a hydrogen bond between Arg330 and the main-chain oxygen of C3/C3b Gly1660. In the TSR6 stirrup, Lys427 forms a salt bridge with C3/C3b Glu1654. This interaction is further stabilized by hydrogen bonds between the TSR6 Glu422 side chain with backbone atoms from Ser1571 and Thr1568 from the C3/C3b-CTC domain and backbone mediated interactions between Val421 and Glu422 from the TSR6 stirrup with C3/C3b Val1657 and Val1658, respectively (Figure 9C).

To gain insights into the properdin interactions with the C3bBb complex, we modeled and refined the structure of the proteolytic fragment Pc in complex with the SCIN-stabilized C3bBb convertase (PDB ID: 5M6W) (32). Modeling properdin in the density of 5M6W (see section Materials and Methods) resulted in a significant improvement of the refinement statistics (Rfree/Rwork = 0.264/0.219, compared to Rfree/Rwork = 0.315/0.262, when not including properdin). The structure comprises two copies of the SCIN stabilized C3bBbPc complex with density for TSR3 only detectable in one copy (Figure 9D). In both copies of the C3bBb-SCIN-Pc complex, the ring-like structure of properdin and the interface with C3b are similar as observed for P^N1/456^ in complex with C3/C3b CTC domain. Although the stirrup loops of TSR5 and TSR6 are in the vicinity of the VWA domain of Bb, we observe only two contacts between properdin and Bb within 3.2 Å in the model. The side chains of Lys350 (325 in 5M6W) of Bb and Val421 of properdin are within 2.8 Å and the side chains Met394 (369 in 5M6W) of Bb and Glu422 of properdin are within 3.1 Å distance, thus no direct interactions are apparent between properdin and Bb in the structural model (Figure 9E).

**Figure 10:**
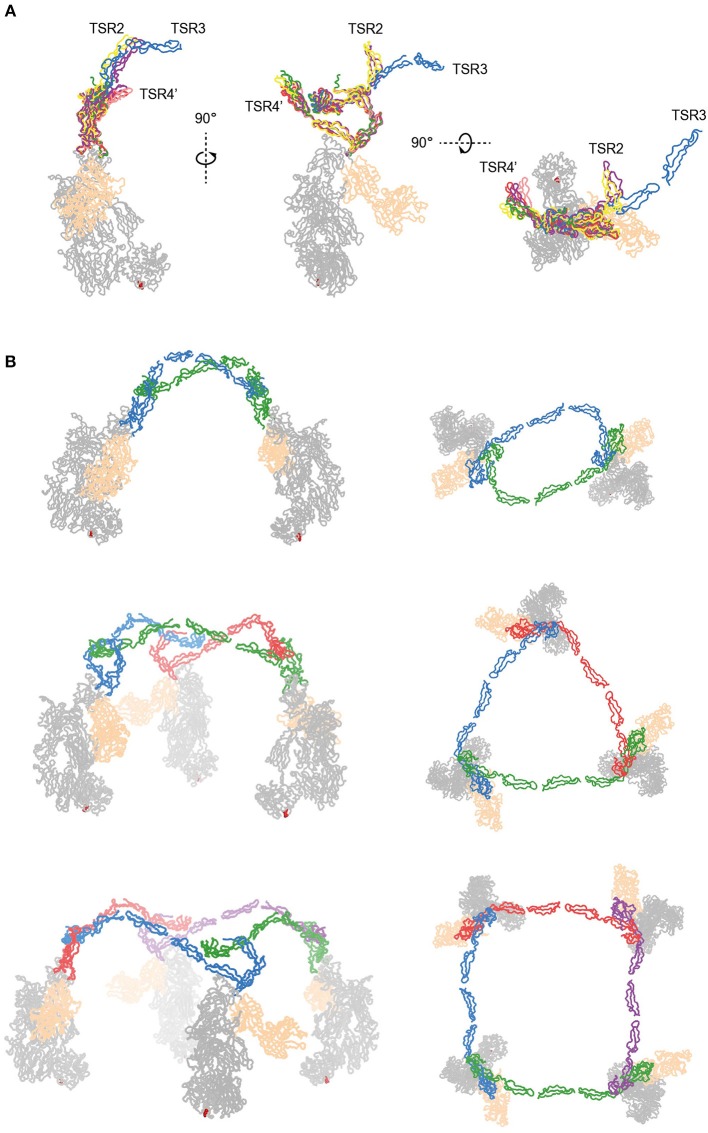
Models of properdin oligomers binding to surface bound C3 convertases. **(A)** Structures of P^N1/456^ (red), P^N12/456^ (yellow), P^N1/456^-CTC (green), and the copy from Pc-C3bBb-SCIN lacking density for TSR3 (pink) superimposed on TSR5 of the other copy of Pc-C3bBb-SCIN (purple). **(B)** Ribbon representation of properdin oligomers binding to C3 convertases viewed from the front (left) and top (right). C3b and Bb are colored gray and wheat, respectively, Gln1013 from the C3b thioester is shown as red spheres. Each protomer in a properdin oligomers is colored differently. Top: Properdin dimer binding to two C3 convertases (for this model we used P^N12/456^ with TSR3 positioned relative to TSR2 as it is in the copy of Pc-C3bBb-SCIN that contains TSR3) Middle: Properdin trimer binding to 3 C3 convertases (for this model the properdin copy from Pc-C3bBb-SCIN that contains TSR3 was used). Bottom: Properdin tetramer binding to four C3 convertases (this model was generated with TSR2 as in the middle panel but using TSR4 from P^N1/456^).

The two C3bBbPc complexes in the asymmetric unit show variation in both TSR4 and TSR2-TSR3; In one of the complexes the conformation of TSR4 is similar to that of TSR4 from P^N1/456^, in the second C3bBbPc complex TSR4 is once again bent at the position of V266, but at an angle that does not correspond to TSR4 in P^N1/456^, P^N112/456^, or P^N1/456^-CTC, showing that TSR4 has an even greater range of motion. This structural variability of the TSR4 conformation results in a ~60° that is covered by TSR4 in all properdin models (Figure 9F). Similarly, TSR2 also shows structural flexibility; the orientation of TSR2 in one C3bBbPc complex matches the orientation observed in P^N12/456^, whereas in the other copy TSR2 is at a 58° angle compared to TSR2 from P^N12/456^ (Figure 10A). Using the different conformations observed for TSR2 and TSR4 we were able to build models for properdin dimers, trimers and tetramers bound to a C3bBb coated surface (Figure 10B). In these models, the properdin ring-like vertices (comprising STB, TSR1 and (the distal end of) TSR4′, TSR5′, and TSR6′ (with domains from a second protomer indicated by an apostrophe), are orientated perpendicular to the plane of the surface, with the edges comprising TSR2, TSR3 and the proximal part of TSR4 roughly parallel to the surface.

## Discussion

Previous biochemical data (11, 12, 21) has indicated that properdin enhances complement activity by binding and stabilizing surface-bound C3 pro-convertases (C3bB) and convertases (C3bBb) of the alternative-pathway. Low-resolution structural data suggested that properdin binds C3 convertases at the α′-chain of C3b (13, 32), consistent with stabilization through putative bridging interactions between C3b and FB or fragment Bb of the pro-convertase and convertase, respectively. The crystallographic data presented here has provided atomic models of the ring-shaped structures previously observed in low-resolution EM images of full-length oligomeric properdin (13, 27) and in a crystal of C3bBb-SCIN in complex with the proteolytic Pc fragment at 6-Å resolution (32). Our high-resolution data reveals the STB-domain fold adopted by the N-terminal domain, the structural variations and post-translational modifications present in the TSR domains and the non-covalent binding interfaces between N-terminal domains STB and TSR1 and C-terminal domains TSR4 and TSR6, respectively, of two different protomers needed to form the ring-shaped structures of properdin. Next, our data of properdin in complex with the CTC domain of C3b shows the interaction details that position properdin on top of a C3b molecule, when C3b is covalently bound to a target surface, and identified two “stirrup-like” loops, formed by inserts into TSR-folds of TSR5 and TSR6, as interaction sites for binding the VWA domain of FB and Bb for stabilizing the C3 pro-convertase and convertase, respectively.

Mass spectrometry of plasma-derived full-length properdin indicated complete C-mannosylation of 14 out of the 17 tryptophans present in the WRWRWR motifs and no or partial C-mannosylation of the remaining three (Trp80, 202, and 318), in addition to three fully (Thr151, Ser208, and Thr272) and one partially occupied (Thr92) O-fucosylation sites and a single N-linked glycosylation site (Asn428) (34, 35) (Figure 4B). We observed that the C-mannosyl moieties on tryptophan are part of common H-bonding networks that also include the backbone nitrogen of the mannosylated Trp (positioned on strand A), the guanidium head group of the arginine distal to the Trp (in strand B) and a polar or negatively charged side chain of the residue opposing the Arg (in strand C), thus bridging all three strands providing stabilization to the TSR fold (Figure 6C). Similar arrangements are found in the structure of TSR domains of C8 (PDB ID: 3OJY), C9 (PDB ID 6CXO), ADAMTS13 (PDB ID:3VN4), and Unc5a (PDBID: 4V2A). In the case of Unc5a (determined at 2.4-Å resolution), the two mannosyl moieties have not been included in the model, but are clearly visible in the density in a conformation similar to that observed in properdin. In C6 structures (3T5O, 4E0S, 4A5W), the mannoses in TSR1 and TSR3 domains are absent or modeled in various alternative conformations, possibly due to the relatively low resolution of these structures, ranging from 2.9 to 4.2 Å. In our structures, we observed clear density for all mannosyl moieties, except two (Trp86 and Trp145), of the reported fully C-mannosylated tryptophans (35). Trp145 is located on TSR2, which exhibits overall poor density in the crystal structure of P^N12/456^. Very weak densities for a mannosyl moiety at Trp86 of TSR1 were observed in all three structures. The WRWRWR motif in TSR1 lacks the final arginine residue, instead a glutamine residue is observed at this position. Most likely, the absence of H-bonding potential with a guanidinium moiety at the final position causes local flexibility, explaining the weak density observed for the mannosyl on Trp86. Properdin is N-glycosylated at Asn428 of TSR6, which is located at the base of the β-hairpin insertion. In our structures this glycan is only partially present, however, there is clear density for this glycan in 5M6W. This glycan would not interact with C3bBb upon binding, which is in agreement with previous findings that removal of N-linked glycans had no effect on properdin activity in a hemolytic assay (29). Properdin O-fucosylation is observed in the density at Thr92, Thr151, and Thr272, which are positioned at structural homologous positions in the A-B-loop of TSR1, TSR2, and TSR4. The A-B loop in 63 out of 88 TSR sequences contains the sequence C-X-X-S/T-C, where the serine or threonine is O-fucosylated (60). Similar to TSR1 from C6 and the TSR domain from ADAMTS13, the O-glucosyl-β1,3-fucose is packed against the disulphide bridge that connects loop A-B to the terminal residue of the TSR domain.

Oligomeric full-length properdin consists of ring-shaped vertices, formed by N- and C-terminal domains of separate protomers (13, 27). The crystal structures of P^N1/456^ and P^N12/456^, obtained by co-expression of N- and C-terminal parts, clearly revealed that the ring-shaped vertices are formed by two contact interfaces between N-terminal domains of one protomer and the C-terminal domains of another protomer (Figure 4A). The N-terminal domain adopts a STB fold and binds the TSR4′ domain of another protomer. This interface, which is dominated by hydrophobic interactions, is further stabilized by additional H-bonds between STB Asn59 and the O-glucosyl-β1,3-fucose on Thr272 of TSR4′. A second protomer-protomer interface is observed between TSR1 and TSR6′. This interface is formed between the distal end of TSR6′ and the β-sheet at the core of TSR1 and involves hydrophobic interactions as well as several H-bonds and two salt bridges. Overall, the ring-shaped vertex of properdin is formed by STB-TSR1 of one protomer and (approximately ~1/3 of) TSR4′, TSR5′ and, an extended and curved, TSR6′ of a second protomer (Figure 8). TSR2, TSR3, and the remaining part of TSR4 consequently form the edges in properdin oligomers.

Consistent with low-resolution EM and X-ray data (13, 32), we have shown that the TSR5 domain of properdin provides the main interaction interface with C3b by binding along the length of the C-terminal α-helix of the C3b α′-chain (Figure 9A). Protonation of properdin His369, at this main interface, would yield formation of a salt-bridge with C3b Glu1654 (Figure 9C), explaining increased binding of properdin to C3b at low pH (32, 62). Comparison with other structures of C3b (37) indicates that binding of properdin to the CTC domain does not require nor likely induces large conformational changes in C3b. We identified two “stirrup”-like loops, residues 328–336 of TSR5 and 419–426 of TSR6, which embrace the end of the C-terminal α-helix of CTC (Figure 9C). Cleavage of properdin in the TSR5-stirrup loop (between res. 333–334) leads to loss of C3b binding (and, hence, loss of convertase stabilization) (29), which indicates the importance of an intact TSR5 stirrup in C3b binding. The only known properdin type III (loss-of-function) mutation, Y414D (63), is located at the base of the TSR6 β-hairpin that constitutes the TSR6 stirrup. Tyr414 is part of a hydrophobic core between TSR5 and TSR6 (Figure 7) and Y414D likely disturbs this hydrophobic core and destabilizes the TSR6 stirrup and hence affects C3b binding or convertase stabilization (63).

Monomerized properdin binds the C3 convertase (C3bBb) and pro-convertase (C3bB) strongly, and C3b weakly (K_D_′s of 34 nM, 98 nM, and 6.8 μM, respectively, in agreement with previous data (12, 32); Figures 2, 3). Superposition of P^N1/456^-CTC onto C3bB and C3bBD (PDB ID: 2XWJ and 2XWB) (39) suggests that the two stirrups are ideally positioned to bridge interactions between C3b and the VWA domain of FB and Bb. The TSR5 stirrup is in close proximity to the N-terminal region of CCP1 in the Ba region of FB, with only one potential H-bond between properdin Asn331 and FB Ser78. The proximity of properdin to FB-CCP1 explains the cross-links observed between Ba and properdin by Farries et al. (64). Re-analysis of C3bBb-SCIN with Pc (at 6-Å resolution) is consistent with the interactions that we observed at high resolution between P^N1/456^ and an isolated C3/C3b-CTC domain (Figures 9D,E). The low-resolution data of Pc-C3bBb-SCIN suggests small rearrangements in the TSR6 stirrup loop. Nevertheless, the expected additional interactions between Bb and properdin are not observed in Pc-C3bBb-SCIN. Potentially, the inhibitor SCIN enforces a C3bBb conformation that is not compatible with stabilization by properdin (32). Therefore, the interaction details between properdin and FB and Bb that explain higher binding affinities for the pro-convertase and convertase remain unfortunately unresolved.

**Figure 11:**
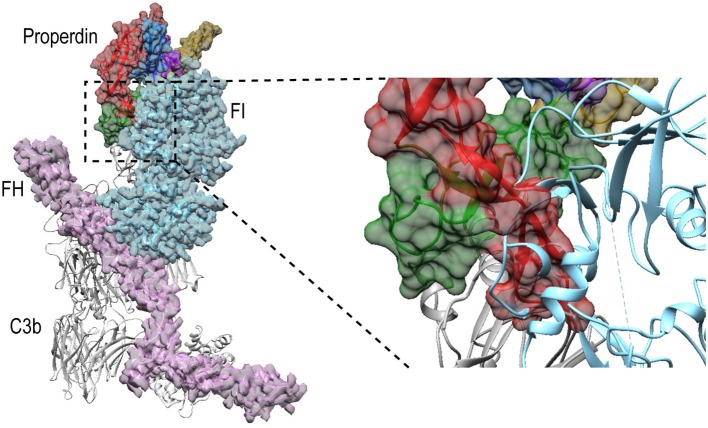
Properdin binding to C3b is incompatible with FI binding. Superposition of P^N1/456^-CTC and C3b-FH-FI (PDB ID: 5O32). Models were superposed on the C3b-CTC domains (rmsd 0.7 Å). Left: overview of the structures with FH (Pink), FI (light blue), and properdin (multicolored model, with TSR5 in green and TSR6 in red) in ribbon presentation with semi-transparent molecular surface and C3b (gray) shown in ribbon. Right: close up showing FI occupies the same space as the properdin TSR6 (red) stirrup loop.

Besides promoting the formation of, and stabilizing the alternative-pathway C3 convertase, properdin is also known to inhibit FI activity (12, 13, 65); based on kinetic data, this is likely due to competition for the same binding site on C3b (12). Superposition of P^N1/456^-CTC with C3b in complex with FH and FI (66) (PDB ID: 5O32) shows that, in a putative properdin-C3b-FH-FI complex, TSR6 of properdin severely clashes with the FI membrane-attack complex domain in FI (Figure 11). Therefore, the structural data supports competitive binding of properdin and FI for the same binding site. No overlaps are observed between properdin and regulators FH, DAF and MCP, when superposing P^N1/456^-CTC with other C3b-regulator complexes (37). Thus, reduced decay-acceleration activity of FH and DAF (32) is most likely due to the increased stability of C3bBb upon properdin binding.

Native properdin occurs predominantly as a mixture of dimers, trimers and tetramers (8), observed as flexible lines, triangles and quadrilaterals in negative-stain EM (13, 27). The oligomers bind with high avidity (with an apparent K_D_ of 22 nM) to surface-bound C3b compared to monomerized properdin binding a single C3b (K_D_ of 6.8 μM). Consistently, properdin tetramers are more active than trimers, which are more active than dimers (8, 9). In the structures presented here, overlaid in Figure 10A, we observed structural variability predominantly in TSR2 and TSR4. These variations occur mostly in the plane of the membrane of a properdin oligomer bound to an opsonized surface, which allowed us to create composite models representing symmetric properdin dimers, trimers and tetramers binding to surface-bound C3b, C3bB, or C3bBb in a straightforward manner (Figure 10B). The ability of properdin to form flexible oligomers is crucial to enhance complement activation only on surfaces by binding deposited C3b molecules with high avidity, while promoting convertase formation (11) and stabilizing formed convertases by binding C3bB and C3bBb complexes with high affinity (12, 32). Local production of properdin by immune cells would result in further enhancement near affected sites (23, 25, 26).

## Data Availability

The datasets generated for this study can be found in the RSCB Protein Data Bank with PDB IDs 6S08, 6S0A, and 6S0B.

## Author Contributions

RB and PG designed the project. RB and JG cloned the constructs. RB carried out protein purification, crystallization, and biochemical assays. RB and NP collected and processed diffraction data, determined, and refined structures. RB, TB, and PG analyzed the data. RB, TB, and PG wrote the manuscript.

### Conflict of Interest Statement

The authors declare that the research was conducted in the absence of any commercial or financial relationships that could be construed as a potential conflict of interest.

## Abbreviations

ADP: atomic displacement parameters
Bb: cleavage product b of factor B
C3: complement component 3
C3b: cleavage product b of complement component 3
CTC: C-terminal C345c
DAF: decay-accelerating factor
EM: negative-stain electron microscopy
FB: factor B
FH: factor H
FI: factor I
IMAC: immobilized metal affinity chromatography
MCP: membrane-cofactor protein
Pc: cleaved properdin
PDB: protein data bank
rmsd: root-mean square deviation
SCIN: *Staphylococcus aureus* inhibitor
SEC: size exclusion chromatography
SPR: surface-plasmon resonance
STB: short transforming-growth factor B binding protein-like
TSR: thrombospondin type-I repeat.
